# LungCNET: A High-Performance Deep CNN Model for Lung Cancer Detection Evaluated Against Widely Used CNN Benchmarks

**DOI:** 10.3390/bioengineering13080931

**Published:** 2026-08-18

**Authors:** Elham Eskandarnia, Peter Adepoju, Kaveh Kiani, Taha Mansouri, Ayah Binrajab

**Affiliations:** 1 Faculty of Computer Studies, Arab Open University, Building 890, Street 3220, A’ali 732, Bahrain; 2 Data Science and AI HUB, University of Salford, Manchester M5 4WT, UK; p.a.adepoju@edu.salford.ac.uk (P.A.); k.kiani@salford.ac.uk (K.K.); t.mansouri@salford.ac.uk (T.M.); 3 School of Science, Engineering & Environment, University of Salford, Manchester M5 4WT, UK; 4 School of Medicine, Royal College of Surgeons in Ireland–Medical University of Bahrain, Building 2441, Road 2835, Busaiteen 228, Bahrain; 24200305@rcsi.com

**Keywords:** transfer learning, convolutional neural network, computer vision, computer-aided diagnosis, lung cancer, medical imaging, IQ-OTH/NCCD dataset

## Abstract

Lung cancer arises from mutations in lung cells, disrupting their normal growth cycle and leading to uncontrolled cell division. These rapidly dividing cells lose function and fail to form healthy lung tissue. Several factors contribute to the difficulty of diagnosing and classifying lung nodules, including the high degree of morphological heterogeneity and overlapping characteristics between benign and malignant nodules. Recently, deep learning models have been used in computed tomography (CT)-based lung nodule diagnosis and have demonstrated diagnostic efficiency comparable to that of radiologists. This study introduces LungCNET, a high-performance multi-layer deep convolutional neural network trained on chest CT images to improve lung lesion classification efficiency and accuracy significantly. The data for the Lung Cancer convolutional neural network (LungCNET) were derived from the IQ-OTH/NCCD CT scan dataset (1097 images from 110 cases), split into training (767 images), validation (109) and a held-out test partition (221) that played no role in training or model selection. This dataset encompasses three diagnostic categories: benign, malignant, and normal lung tissues. LungCNET was evaluated against fine-tuned benchmark models that are both established and widely used, spanning architectures introduced between 2014 and 2024, including VGG16, ResNet50, InceptionV3, MobileNetV2, and YOLOv11. On the held-out test partition, LungCNET reached a macro-averaged F1-score of 95.19%, with VGG16 at 94.09% and InceptionV3 at 92.28%; these three models performed comparably, and the separation between them is small relative to the resolution of a test set of this size. LungCNET was, however, the only model to exceed 90% F1-score across all three diagnostic classes simultaneously, and recorded the highest F1-score on the benign class (91.0%), the smallest and most frequently misclassified category, where two of the six models failed entirely. These results support LungCNET as a candidate tool for lung cancer diagnosis, subject to validation on larger and independently sourced datasets.

## 1. Introduction

Lung cancer is a malignant disease marked by uncontrolled cell growth in the lung. It is the most common cause of cancer-related death worldwide, with around 1.8 million deaths per year [1,2]. Its fatality rate exceeds that of most other malignancies. Better approaches to diagnosis and treatment are needed urgently [3]. Two types exist: non-small cell lung cancer, 80–85% of cases, and small cell lung cancer, rarer but more dangerous [4]. Survival chances rise sharply with early detection. Symptoms, though, rarely appear before the disease has already advanced. Routine screening for high-risk groups matters for exactly this reason [2,5,6]. This study focuses specifically on computed tomography (CT), the imaging modality most widely used for lung nodule assessment, and develops a deep learning model trained and evaluated on chest CT images. To distinguish malignant from benign lung nodules, clinicians have traditionally combined imaging modalities, chest X-rays and CT scans, with biopsy [7,8]. CT scans produce detailed cross-sectional images that let clinicians compare nodules by size, shape, and density [9]. This is not easy. Lung nodules vary too much in shape and character, and the same scan read by different radiologists can produce different findings [10]. CT scans are also expensive, adding to the load on healthcare systems already stretched thin [11]. Between the cost and the disagreement in reading them, early diagnosis matters even more [12]. This is what has generated interest in automatic diagnosis methods. Early attempts at automating this process extracted hand-crafted features directly from CT images, texture, geometry, and intensity values to build classification models. These features could separate benign from malignant nodules to a point, but the same subjectivity problem crept back in: nodules varied too much, and models built this way struggled to hold up consistently across cases [13]. Later work tried support vector machines [14,15] and random forests [16], but the same complexity and variability in medical imaging data limited these approaches too. CNNs changed this. Rather than needing features specified by hand, they learn directly from the data, closing much of the gap that limited earlier machine-learning approaches and reaching diagnostic effectiveness comparable to radiologists [8,17,18,19]. Setio et al. built a multi-view computer-aided detection system for pulmonary nodules and reported 90.1% sensitivity on the LIDC-IDRI dataset [20]. The same architecture family performs well outside oncology too: a VGG16-based model detected COVID-19 pneumonia from chest X-ray images with 98.1% accuracy [21]. Public datasets like LIDC-IDRI [22] and the National Lung Screening Trial (NLST) [23] have since supported a large body of work on automated nodule classification.

LungCNET targets the harder three-class problem, benign, malignant, normal, rather than the binary cancerous/non-cancerous split most prior work uses. Dropout regularisation and a carefully sized convolutional structure control overfitting on the comparatively small dataset. The aim is a model fast enough at inference for real clinical use and robust to the noise real CT scans carry.

### Contribution of the Research

This research addresses the main challenges in lung cancer diagnosis, particularly the diagnostic value of early detection given the disease’s severity. We propose a new multi-layered convolutional neural network, LungCNET, designed specifically for lung lesion classification. Evaluated on a held-out test partition, LungCNET reached a macro-averaged F1-score of 95.19% across the three classes, ahead of VGG16 (94.09%), InceptionV3 (92.28%), YOLOv11 (71.29%), MobileNetV2 (60.71%) and ResNet50 (49.63%). The margin over the strongest fine-tuned benchmarks is narrow, and LungCNET is best characterised as performing comparably to them rather than decisively better. Its distinguishing property is consistency: it is the only model tested to exceed 90% F1-score in all three diagnostic categories at once, and it performs best on the benign class, which the other architectures classify least reliably.

## 2. Previous Work

False positives and overdiagnosis remain unresolved despite better screening technology [3]. Classification improvements offer one route to closing that gap.

GoogLeNet, fine-tuned by Al-Huseiny and Sajit on the IQ-OTH/NCCD dataset, reached 94.38% accuracy [24]. Most of that gain came from a single pre-processing step: extracting the lung region before training, rather than from the architecture itself.

Pandian et al. ran a head-to-head comparison instead, GoogLeNet against VGG-16, on adenocarcinoma, large cell carcinoma, and squamous cell carcinoma versus normal tissue. VGG-16 won, 98% accuracy at the image level [25].

A different route entirely: Teramoto et al. built a shallow DCNN, three convolutional layers, three pooling, two fully connected, and trained it on cytological images directly, skipping CT altogether. 71% correct classification. Roughly matching trained cytotechnologists [26].

Heuvelmans et al. took the external-validation route. Their Lung Cancer Prediction CNN (LCP-CNN), trained on NLST data, then validated against the separate LUCINDA cohort, came out at 94.5% AUC and 99.0% sensitivity [27].

Solyman and Schwenker went the ensemble direction. VGG-16 by itself: 89% test accuracy. Adding ResNet50, InceptionV3, and EfficientNetB7 through majority voting pushed it to 92.8% [28]. Table 1 summarises all five.

None of these five targets the same three-class problem LungCNET does. Most separate cancerous from non-cancerous tissue; ours also separates benign from malignant, within one pipeline.

LungCNET’s architecture was built specifically for this task rather than adapted through transfer learning. Dropout layers and a carefully sized convolutional structure control overfitting on a comparatively small dataset, without leaning on features learned from unrelated image domains.

Ensemble approaches like Solyman and Schwenker’s combine VGG-16 [29], ResNet50 [30], and InceptionV3 [31] at real computational cost. LungCNET targets comparable accuracy from a single network rather than an ensemble.

## 3. Methodology

### 3.1. CNN Benchmark Models

We compare LungCNET against five widely used transfer learning models: VGG16, ResNet50, InceptionV3, MobileNetV2, and YOLOv11 [32]. Each was pre-trained on large image datasets, then fine-tuned on our CT scan dataset for lung cancer classification. These five were selected because each has a pre-trained implementation available in the same framework used throughout this study (tf.keras.applications for the four CNN backbones, and the Ultralytics library for YOLOv11), allowing a controlled, reproducible comparison under identical training conditions. GoogLeNet (Inception-v1) and LCP-CNN, cited in Table 1 as prior results reported by other groups on their own datasets and splits, were not reimplemented here for the same reason: GoogLeNet has no pre-trained implementation in tf.keras.applications, unlike the other four CNN backbones, and would require sourcing weights from a different framework entirely; LCP-CNN’s architecture and trained weights are not publicly released.

We unfroze the final layers of each benchmark model so it could adapt to this task while keeping its general pre-trained features. The number of unfrozen layers varied by architecture, enough to allow adaptation without inviting overfitting. Training used exponential decay scheduling, early stopping, and learning rate reduction to keep convergence efficient and avoid overfitting.

### 3.2. LungCNET Model

**Figure 1:**
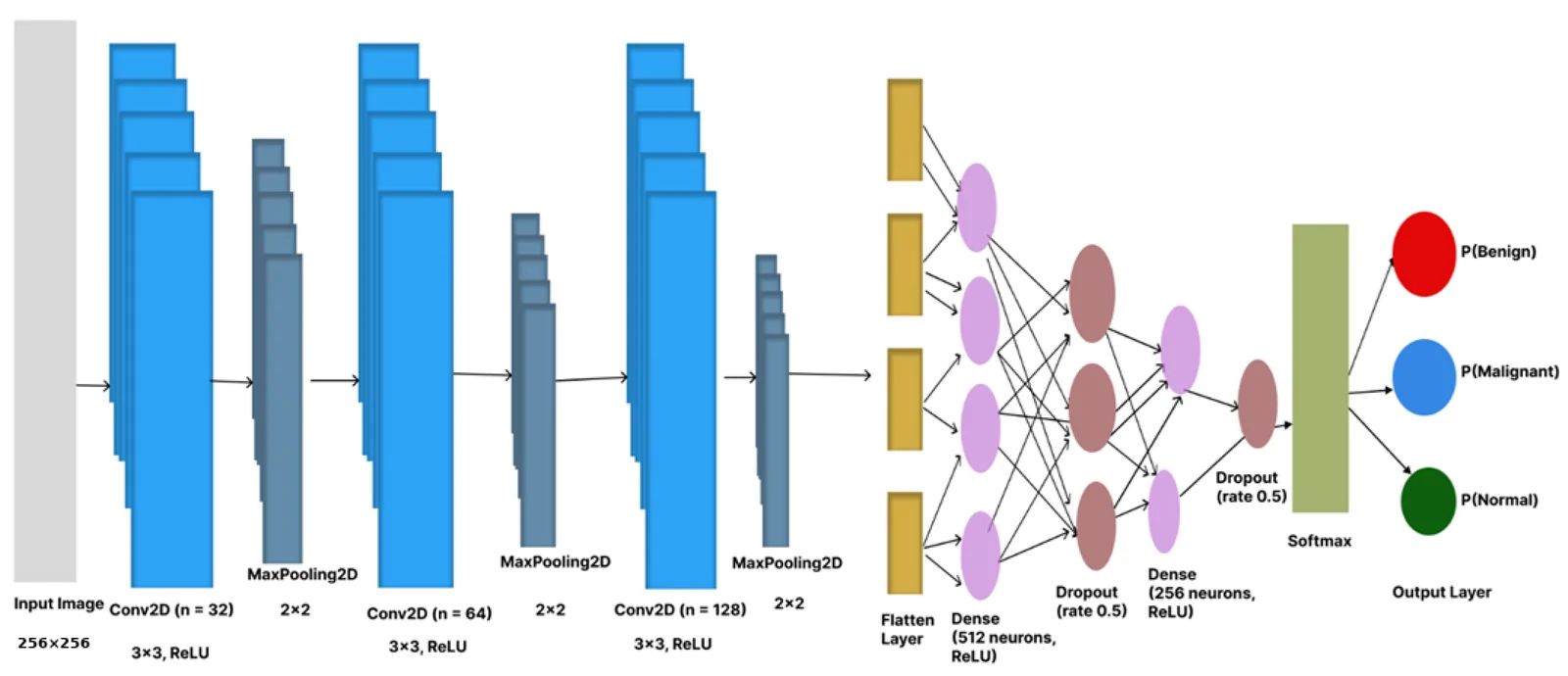
LungCNET architecture. The model takes 256×256 input images through three convolutional blocks (Conv2D with ReLU activation, each followed by MaxPooling2D), then flattens and uses dense layers with dropout, ending in a three-class softmax output layer for Benign, Malignant, and Normal classification.

LungCNET is a custom CNN built specifically for lung cancer classification. It sorts CT scan images into three classes: benign, malignant, and normal. The architecture is sequential: convolutional layers, max-pooling, fully connected layers, and dropout for regularisation (Figure 1).

Three convolutional layers open the network, with filter depth increasing from 32 to 64 to 128, each using ReLU activation and followed by max pooling to downsample the feature maps. After the convolutional blocks, the data is flattened and passed through two fully connected layers of 512 and 256 neurons. Each carries a dropout layer at a rate of 0.5 to reduce overfitting. A softmax activation on the output layer produces the probability distribution across the three classes.

We trained LungCNET with the Adam optimiser and categorical cross-entropy loss. Its multi-layer architecture learns hierarchical features directly from the CT scans.

We built LungCNET from scratch for this task, rather than adapting an existing model. The convolutional and dense layers are configured to capture the spatial characteristics specific to lung CT scans, rather than reusing features learned from unrelated natural-image datasets. Dropout and ReLU activations balance regularisation against non-linearity during training, and we tuned hyperparameters alongside the Adam optimiser. Early stopping and TensorBoard callbacks kept training efficient and let us monitor performance in real time.

Training the architecture from scratch, rather than repurposing a pre-trained backbone, means the model learns only features present in this CT data, with regularisation chosen for a dataset of this size. Section 4 reports how this compares against the fine-tuned benchmarks.

### 3.3. Dataset Composition and Class Distribution Analysis

**Figure 2:**
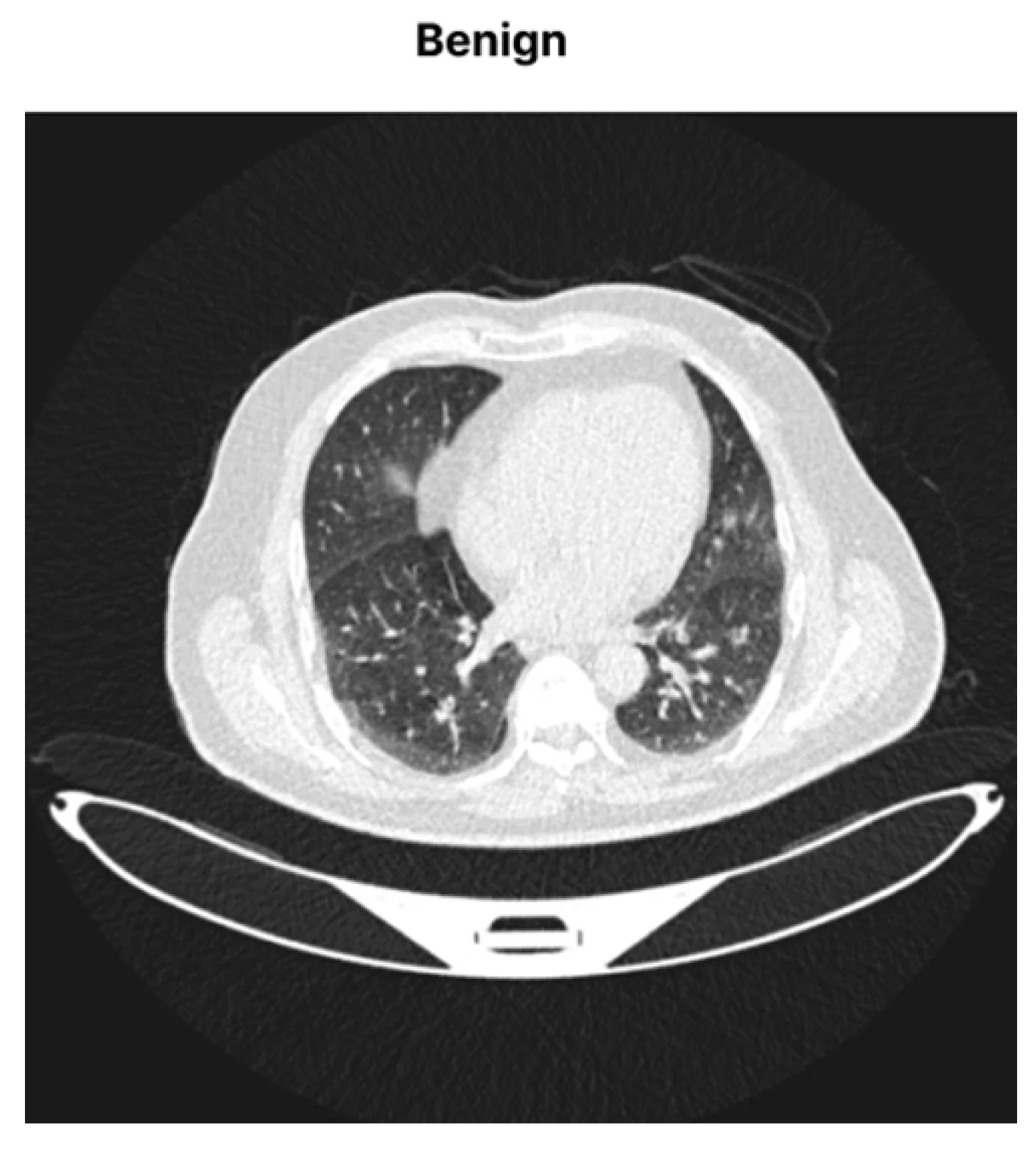
Sample CT scan showing a benign lung nodule.

**Figure 3:**
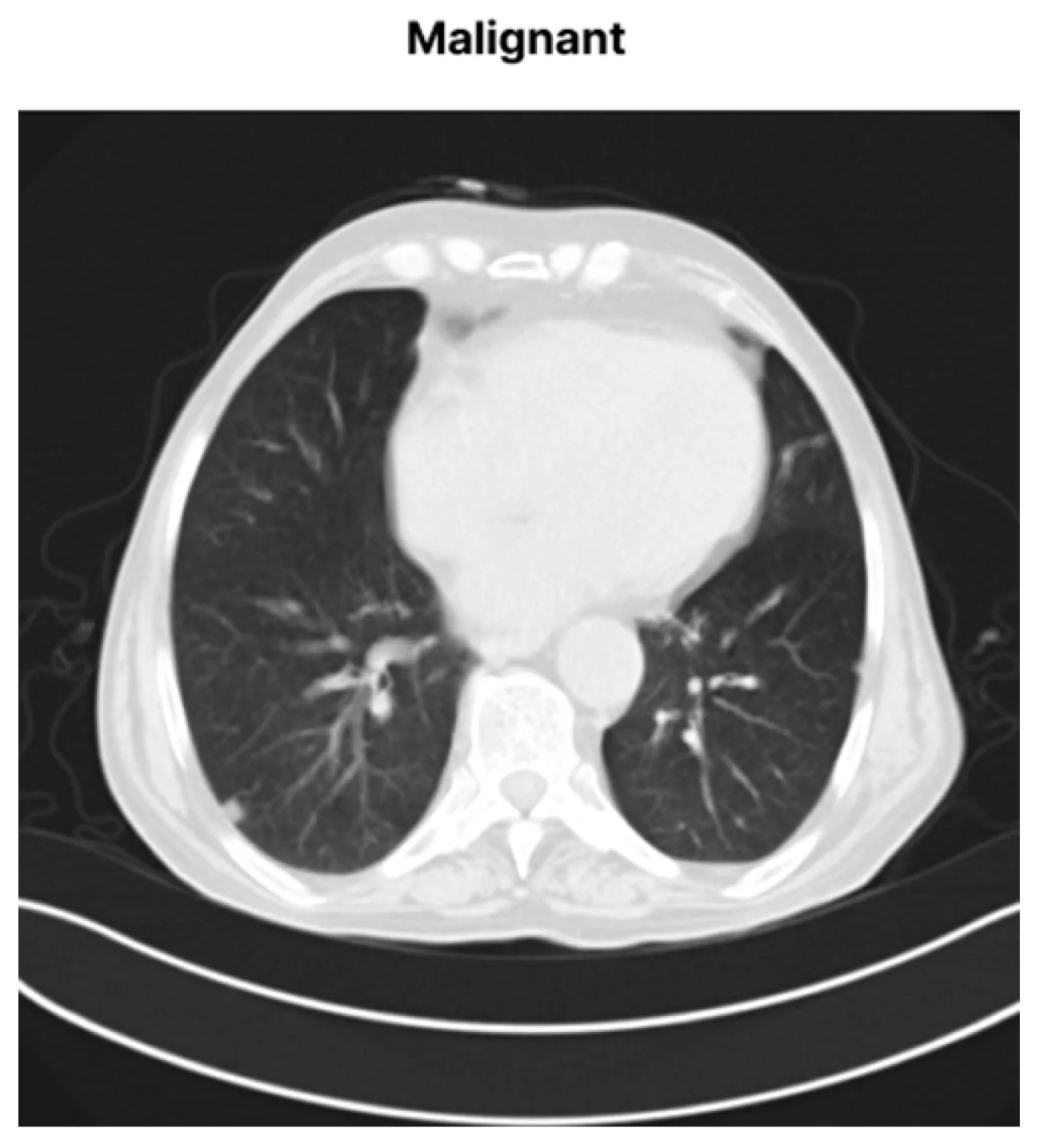
Malignant CT scan image sample.

**Figure 4:**
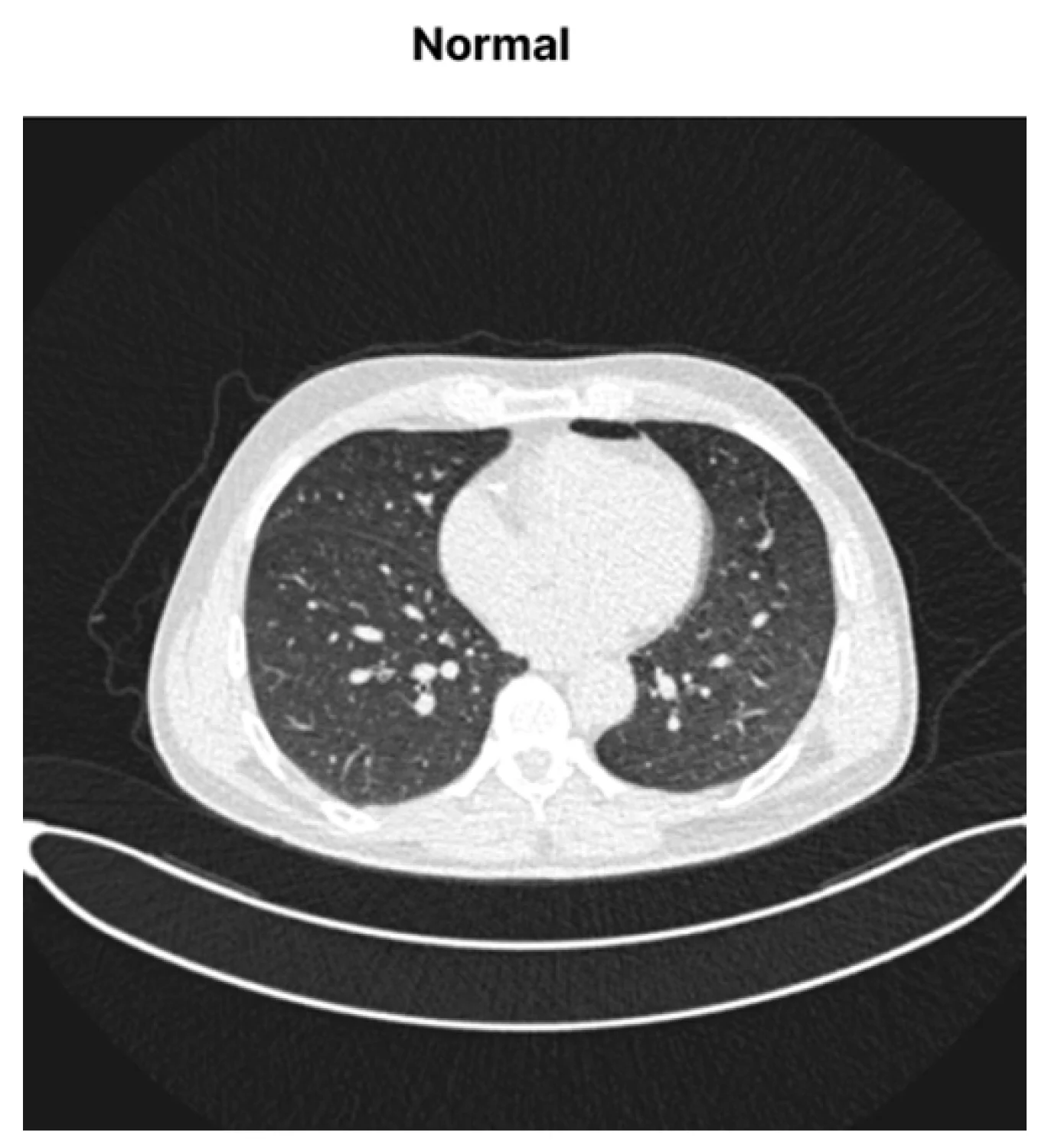
Sample CT scan of normal lung tissue.

We used the IQ-OTH/NCCD lung cancer dataset [33]: 1097 CT scan images from 110 distinct cases, split into three categories—benign (Figure 2), malignant (Figure 3), and normal (Figure 4). The dataset comes from the Iraq-Oncology Teaching Hospital/National Center for Cancer Diseases and covers a range of patient demographics and cancer stages. Of the 1097 images, 767 (70%) were allocated to training, 109 (10%) to validation, and 221 (20%) to a held-out test partition, as detailed in Section 3.

**Figure 5:**
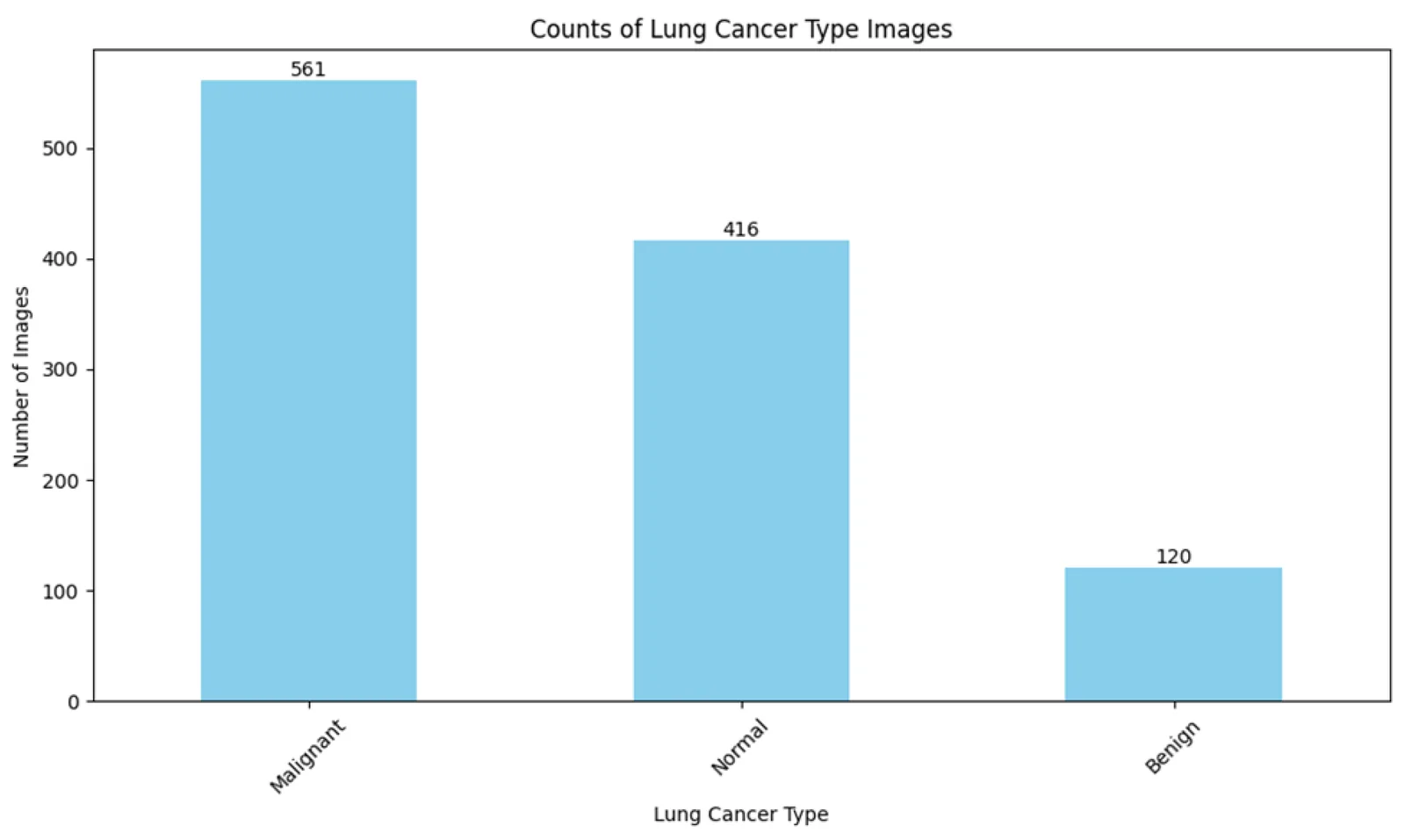
Distribution of lung nodule classes in the IQ-OTH/NCCD dataset: number of images per class (benign, malignant, normal), showing the under-representation of the benign class.

Figure 5 shows the distribution across the three lung nodule classes. The bar chart makes the dataset’s class imbalance easy to see; benign is markedly under-represented, which is addressed through augmentation and reflected in the per-class results reported in Section 4.

We addressed this imbalance and improved the model’s generalisation to unseen data through data augmentation applied to the training set only. Using TensorFlow’s tf.image operations, images were randomly flipped horizontally during training, which reduces the model’s sensitivity to left/right orientation without altering anatomy in ways that do not occur in real scans; left/right chest laterality carries no diagnostic weight for the benign/malignant/normal classification task here. The dataset was split into three partitions using scikit-learn’s train_test_split with random_state=42, stratified by class so that each partition preserves the overall benign/malignant/normal proportions: 70% for training (767 images), 10% for validation (109 images), and 20% as a held-out test partition (221 images). The validation partition is used only for early stopping and learning-rate scheduling during training. The test partition is used once, after all training is complete, and plays no part in training, model selection or hyperparameter choice; all performance figures reported in Section 4 are computed on it. The same three partitions were used for every model, so the comparison across architectures is like-for-like. As noted in Section 7, this split is image-level rather than patient-level, because the public release of the dataset does not provide slice-to-patient mapping.

## 4. Models Implementation and Performance Analysis

To evaluate the models’ performance, the training, fine-tuning, and validation processes of each model are first examined. The models’ performance is then compared on the held-out test partition (see Section 3 for the split), and their classification decisions are visualised using confusion matrices.

### 4.1. Training, Fine-Tuning, and Validation Comparison Analysis of the Models

**Figure 6:**
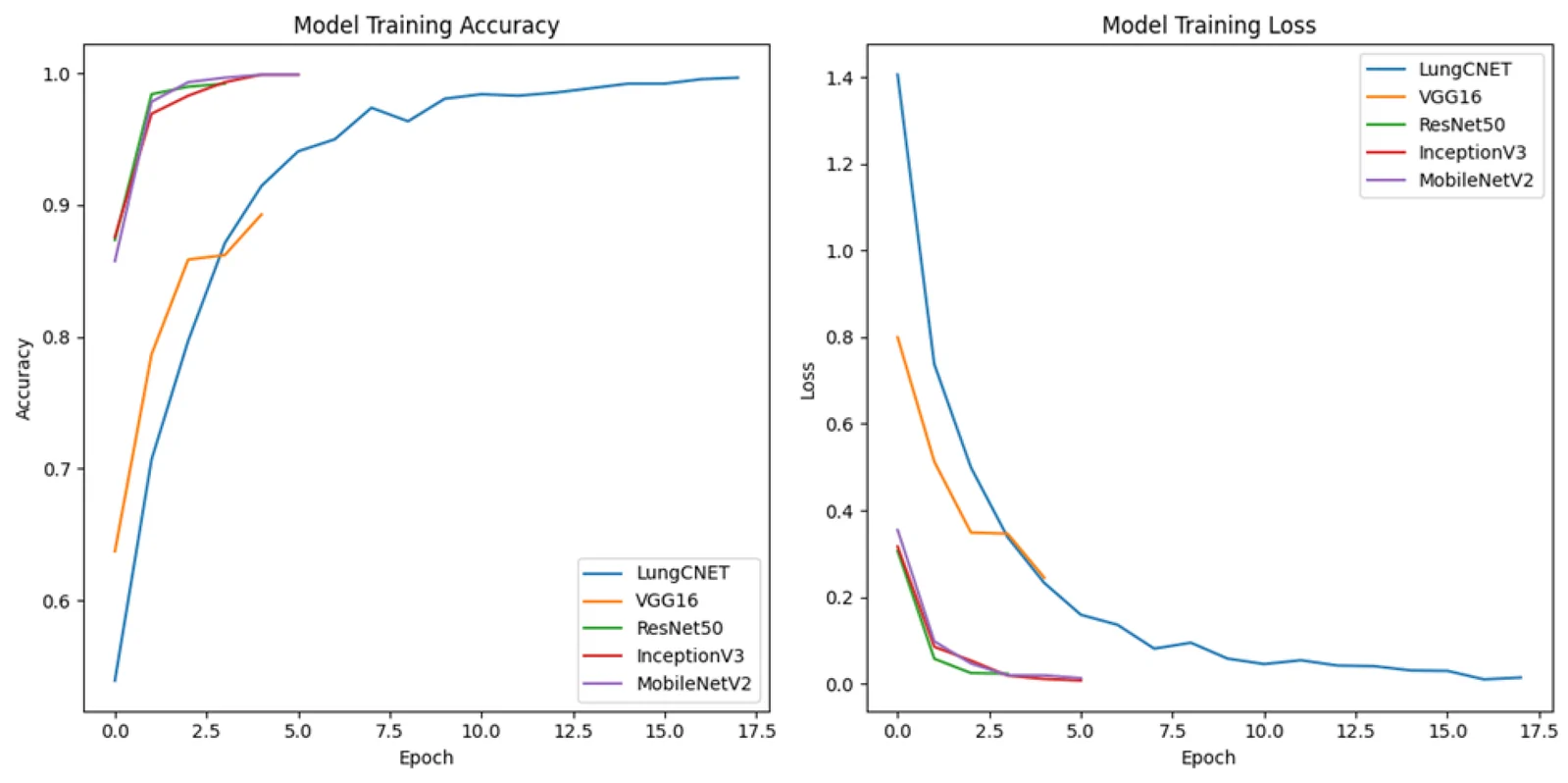
Training convergence for LungCNET, VGG16, ResNet50, InceptionV3 and MobileNetV2, recorded during the initial training runs under the earlier 80/20 configuration. Illustrative of convergence behaviour in a single run; see Section 7 on run-to-run variation.

**Figure 7:**
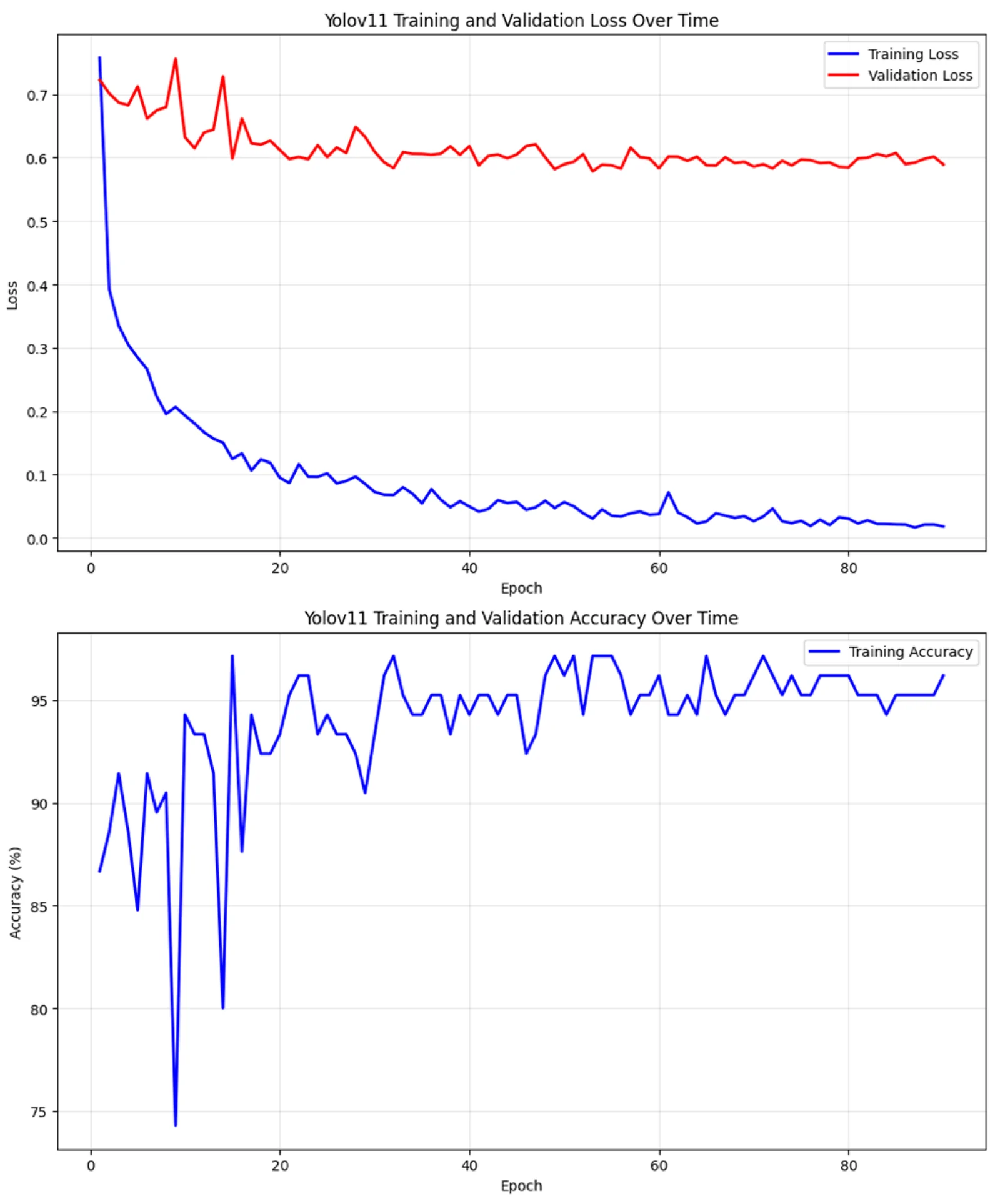
Training convergence for YOLOv11, recorded during the initial training runs under the earlier 80/20 configuration. Illustrative of convergence behaviour in a single run; see Section 7 on run-to-run variation.

Table 2 and Figure 6 and Figure 7 report convergence behaviour observed during the initial training runs of this study. They are retained as a description of how each architecture learns from this data, and are not the study’s performance results: all reported performance is computed on the held-out test partition and given in Section 4. Two caveats apply to reading them. First, they were recorded under the earlier 80/20 train/validation configuration rather than the three-way split adopted here, so the validation figures are not directly comparable with the test-partition results in Table 3, Table 4, Table 5 and Table 6; validation accuracy runs systematically higher than test performance for every model. Second, as discussed in Section 7, repeated training runs of the fine-tuned benchmarks produced substantially different convergence behaviour, so these trajectories should be read as illustrative of one run rather than characteristic of each architecture.

### 4.2. Comparative Analysis of Models’ Performance on the Test Dataset

Table 3 reports benign classification, the minority class in this dataset (24 of 221 test images). LungCNET reached the highest F1-score at 91.0% (100.0% precision, 83.3% recall), followed by VGG16 at 85.7% and InceptionV3 at 82.6%. YOLOv11 reached 27.0%. ResNet50 and MobileNetV2 both scored 0% across all three metrics, failing to predict the benign class at all. This minority-class collapse is consistent with the fine-tuning setup described in Section 3: fine-tuning a large pre-trained network on a small, class-imbalanced dataset is a known failure mode in which the model defaults to the majority classes and never predicts the minority class. It is corroborated by Table 6, where these same two models record the lowest macro-averaged F1-scores of the six tested. Because the benign partition contains only 24 images, individual predictions carry substantial weight: one image corresponds to approximately 4.2 percentage points of recall, and the difference between LungCNET and VGG16 on this class amounts to two images (20 of 24 versus 18 of 24). The 95% Wilson confidence intervals for these two recall figures, [64.1%, 93.3%] and [55.1%, 88.0%] respectively, overlap substantially, and the ranking between them should not be treated as firmly established on a test set of this size.

**Table 3:** Performance metrics comparison for benign class classification.

Model	Precision	Recall	F1-Score
LungCNET	100.0%	83.3%	91.0%
VGG16	100.0%	75.0%	85.7%
InceptionV3	86.4%	79.2%	82.6%
YOLOv11	67.0%	17.0%	27.0%
ResNet50	0.0%	0.0%	0.0%
MobileNetV2	0.0%	0.0%	0.0%

For malignant classification (Table 4), the largest class in the test partition (113 of 221 images), performance was uniformly high. VGG16 reached 100.0% precision and recall, InceptionV3 99.6% F1-score, and LungCNET and YOLOv11 both 99.0%. MobileNetV2 reached 97.3%. ResNet50 trailed at 81.8%, its high recall (97.3%) offset by low precision (70.5%), consistent with a model defaulting to the majority class. The top five models are separated by under three percentage points on this class, which on 113 images corresponds to a small number of individual predictions.

**Table 4:** Performance metrics comparison for malignant class classification.

Model	Precision	Recall	F1-Score
VGG16	100.0%	100.0%	100.0%
InceptionV3	99.1%	100.0%	99.6%
LungCNET	100.0%	97.0%	99.0%
YOLOv11	97.0%	100.0%	99.0%
MobileNetV2	100.0%	94.7%	97.3%
ResNet50	70.5%	97.3%	81.8%

For normal classification (Table 5), VGG16 reached the highest F1-score at 96.6% (93.3% precision, 100.0% recall), with LungCNET close behind at 96.0% (92.0% precision, 100.0% recall) and InceptionV3 at 94.7%. YOLOv11 reached 89.0% and MobileNetV2 84.8%. ResNet50 was weakest at 67.1%, reflecting the same pattern seen in the other two classes.

**Table 5:** Performance metrics comparison for normal class classification.

Model	Precision	Recall	F1-Score
VGG16	93.3%	100.0%	96.6%
LungCNET	92.0%	100.0%	96.0%
InceptionV3	94.1%	95.2%	94.7%
YOLOv11	82.0%	96.0%	89.0%
MobileNetV2	73.7%	100.0%	84.8%
ResNet50	76.9%	59.5%	67.1%

The macro-averaged F1-score gives each class equal weight regardless of its size, providing an overall measure of performance across all three classes. On the held-out test partition (Table 6), LungCNET reached the highest macro-averaged F1-score at 95.19%, followed closely by VGG16 at 94.09% and InceptionV3 at 92.28%. YOLOv11 reached 71.29%, MobileNetV2 60.71%, and ResNet50 49.63%. The 1.10-point separation between the top two models is small relative to what a 221-image test set can resolve, where a single reclassified image shifts overall accuracy by approximately 0.45 percentage points; LungCNET, VGG16 and InceptionV3 are best described as performing comparably rather than as a strict ranking. On macro ROC-AUC the ordering differs: VGG16 records the highest value at 0.9965, ahead of InceptionV3 (0.9877) and LungCNET (0.9799). The clearest distinction between the leading models lies not in aggregate score but in consistency across classes: LungCNET is the only model to exceed 90% F1-score in all three categories simultaneously, and records the highest F1-score on the benign class, which is both the smallest and the most frequently misclassified.

**Table 6:** Macro-averaged F1-score of the three classes for each model.

Model	Macro-Averaged F1-Score	Macro ROC-AUC
LungCNET	95.19%	0.9799
VGG16	94.09%	0.9965
InceptionV3	92.28%	0.9877
YOLOv11	71.29%	0.9666
MobileNetV2	60.71%	0.9354
ResNet50	49.63%	0.8375

### 4.3. Confusion Matrix Analysis of the Models on the Test Dataset

**Figure 8:**
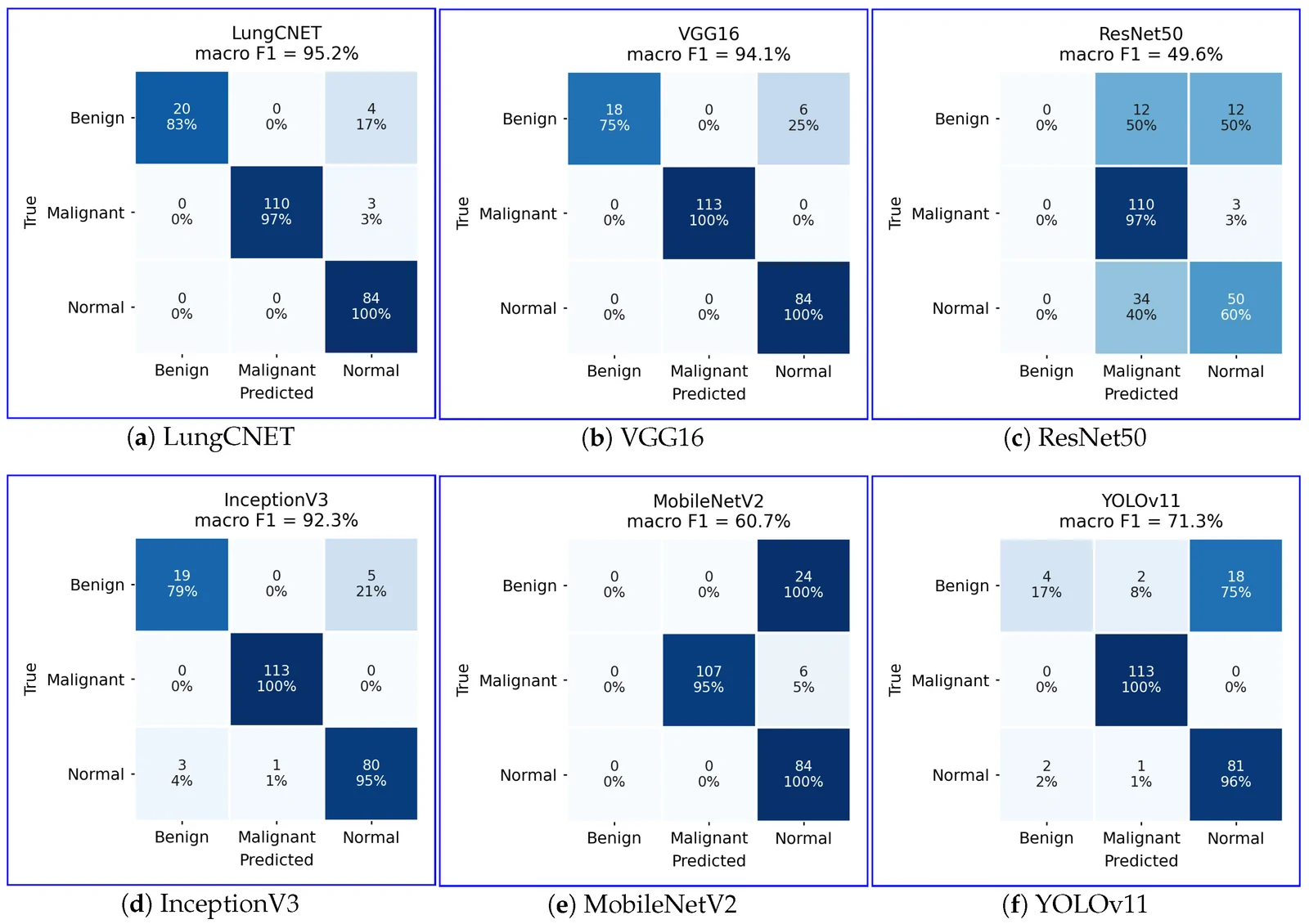
Confusion matrices for all six models on the held-out test partition: (**a**) LungCNET; (**b**) VGG16; (**c**) ResNet50; (**d**) InceptionV3; (**e**) MobileNetV2; (**f**) YOLOv11.

Figure 8 presents confusion matrices for all six models on the held-out test partition. Each cell reports the number of images and the percentage of that true class, so that absolute counts and rates can be read together. The dominant source of error across architectures is the benign class: ResNet50 and MobileNetV2 assign no predictions to it at all, and YOLOv11 recovers only 4 of 24 benign cases, while LungCNET (20 of 24), InceptionV3 (19 of 24) and VGG16 (18 of 24) classify the majority correctly. Benign cases are most often misassigned to the normal class rather than to malignant, which is the less clinically consequential of the two errors. Malignant cases are identified reliably by every model except ResNet50, which recovers 110 of 113 but at the cost of over-predicting that class: 46 images from the other two classes are assigned to it, giving the low precision reported in Table 4.

## 5. Comparison of Computational Efficiency

Table 7 reports two parameter counts for each model: total parameters in the model actually used in this study (pre-trained backbone with the original ImageNet classification head removed, replaced by a single dense layer mapping pooled features to three output classes), and trainable parameters under the fine-tuning setup used here. Rather than freezing the entire backbone, the last several layers of each pre-trained model were unfrozen alongside the new classification head: the last 4 layers of VGG16, the last 10 of ResNet50, the last 20 of InceptionV3, and the last 10 of MobileNetV2. How much of each network this leaves trainable differs considerably between architectures, as set out below the table.

The proportion of each network left trainable varies considerably with the unfreezing configuration. VGG16 trains 7.08 M of its 14.72 M parameters, roughly half, because its unfrozen final four layers include its heaviest fully connected block. ResNet50 trains 4.47 M of 23.59 M and InceptionV3 1.94 M of 21.81 M: although 10 and 20 layers, respectively, were unfrozen, these architectures distribute parameters across many more layers than VGG16, so the unfrozen portion remains a modest fraction of the whole. MobileNetV2 trains 0.74M of 2.26 M, and its overall footprint stays smallest of the pre-trained models because MobileNetV2 is a parameter-efficient architecture by design. YOLOv11 uses the nano-scale YOLO11n-cls architecture with a three-class classification head, totalling 1.53 M parameters [32], all of which were trainable in our setup. LungCNET is the only model with no frozen component: all 59.21M parameters are trainable, which is both the largest total and the largest trainable count of the six.

LungCNET’s total parameter count follows from the layer specification in Section 3: three convolutional blocks feeding a 256 × 256 input into a 512-unit dense layer produces a large flatten step, and the model trains the full 59.21 million parameters that result, from scratch. Its trainable count is the largest of the six models by a wide margin: more than eight times VGG16’s 7.08 M and over thirteen times ResNet50’s 4.47M. This follows directly from training the whole network rather than fine-tuning part of a pre-trained one, and it is the basis for the overfitting concern discussed in Section 6.

Table 8 reports inference latency. LungCNET processes a single image in 55.76 ms and a batch in 95.41 ms, faster than VGG16 (76.21 ms/242.24 ms) and InceptionV3 (110.85 ms/172.14 ms). YOLOv11 is faster still.

Convergence speed varies widely across these architectures, and reflects how much each model needs to learn from this dataset rather than architecture quality on its own: LungCNET trains from scratch, whereas the benchmark models start from pre-trained ImageNet weights and so require less adaptation. Epoch counts are not reported as a comparative measure here, because repeated runs of the fine-tuned benchmarks converged after very different numbers of epochs; this is discussed in Section 7.

## 6. Discussion

On the held-out test partition, LungCNET reached F1-scores of 91.0%, 99.0% and 96.0% on the benign, malignant and normal classes, respectively. Malignant and normal cases were classified reliably by most architectures tested; the benign class separated them. LungCNET recorded the highest benign F1-score (91.0%), ahead of VGG16 (85.7%) and InceptionV3 (82.6%), while ResNet50 and MobileNetV2 failed on this class entirely, and YOLOv11 recovered only 17% of benign cases.

Aggregate performance among the three leading models was close. VGG16 reached 94.09% macro F1 against LungCNET’s 95.19%, and recorded the higher macro ROC-AUC (0.9965 against 0.9799), indicating better-calibrated class separation overall. On a 221-image test set, a single reclassified image moves overall accuracy by roughly 0.45 percentage points, so a 1.10-point separation should not be read as a stable ordering. Two observations nonetheless distinguish LungCNET on this dataset. First, it is the only model to exceed 90% F1-score in all three categories simultaneously, whereas each benchmark has at least one category in which it performs materially worse. Second, it achieves this without pre-trained weights, learning entirely from 1097 CT images, where VGG16 and InceptionV3 begin from ImageNet representations. Whether this consistency reflects a genuine architectural advantage or the particular characteristics of this dataset cannot be settled here, and is the central question for future validation work.

Table 7 shows LungCNET has the largest total parameter count of the six models tested (59.21 M), more than four times VGG16’s 14.72 M. Its practical advantage over the other models is, therefore, not a smaller footprint, but inference latency: at 55.76 ms per image (Table 8) it is the fastest of the five full-scale CNN architectures, ahead of VGG16 (76.21 ms) and InceptionV3 (110.85 ms), since it carries no frozen general-purpose backbone. Only YOLOv11, a nano-scale architecture with 1.53 M parameters, is faster. This raises a related concern worth stating plainly: a model with 59.21 million trainable parameters, trained from scratch on 1097 images from 110 cases, carries a real risk of overfitting to this dataset’s specific characteristics rather than learning generalisable features. Some evidence on this point comes from the corrected evaluation itself: on the validation partition LungCNET recorded a macro ROC-AUC of 1.00, which did not survive evaluation on held-out data, where it fell to 0.9799. The Conclusions set out the further validation needed before these figures should be treated as settled.

## 7. Conclusions

This research introduced LungCNET, a custom convolutional neural network for lung cancer classification. On a held-out test partition it reached a macro-averaged F1-score of 95.19% across the three diagnostic classes, compared with VGG16 (94.09%), InceptionV3 (92.28%), YOLOv11 (71.29%), MobileNetV2 (60.71%) and ResNet50 (49.63%). LungCNET, VGG16 and InceptionV3 performed comparably in aggregate, and VGG16 recorded a higher macro ROC-AUC (0.9965 against 0.9799); the evidence does not support a claim that LungCNET decisively outperforms well-tuned transfer-learning baselines on this dataset. Because LungCNET’s precision and recall stayed high across all three classes rather than only one or two, it may be useful as an assistive tool for radiologists, where consistent performance across diagnostic categories matters more than strength in a single category. This remains to be tested prospectively before any clinical claim can be made. Limitations of this study include the moderate size of the dataset (1097 CT images from 110 cases) and the absence of external clinical validation. Three limitations concerning the evaluation protocol require particular emphasis.

First, the split is image-level rather than patient-level. The public release of the IQ-OTH/NCCD dataset organises images into three class folders with sequentially indexed filenames and provides no metadata linking slices to the 110 source patients described in the dataset documentation. A patient-level split is, therefore, not reproducible from the released files. We contacted the dataset creators to request patient identifiers or a slice-to-patient mapping; should such metadata become available, the experiments reported here can be repeated under a strict patient-level split. The results reported here use a held-out test partition that played no role in training or early stopping, which removes the bias arising from evaluating on the early-stopping partition, but does not exclude the possibility that CT slices originating from the same patient appear in more than one partition. Where slices from one patient are visually similar, this may inflate absolute performance for all six models. The comparison between models remains internally consistent, since every model was trained and evaluated on identical partitions, but the absolute figures should be read as an upper bound.

Second, the test partition is small in the classes that matter most. It contains 24 benign images, so a single prediction corresponds to approximately 4.2 percentage points of benign recall, and differences of a few points between the leading models correspond to one or two images. The reported ranking among LungCNET, VGG16 and InceptionV3 should be treated as provisional on this basis rather than as a settled result.

Third, the reported benchmark figures are single-run point estimates, and repeated training runs indicate that some of them are not stable. Re-running the fine-tuned benchmarks under identical conditions produced substantially different convergence: in one repeat run, MobileNetV2 collapsed onto a single class, its validation accuracy settling at the proportion of that class in the validation partition while validation loss diverged, and ResNet50 reached its best validation loss at the third epoch before deteriorating. LungCNET, VGG16 and InceptionV3 converged consistently across runs. This pattern is itself informative: fine-tuning large pre-trained backbones on a dataset of this size is sensitive to initialisation and batch ordering, which is a plausible contributor to the weak benchmark results reported both here and in the original submission. It also means the ordering among the lower-performing models (ResNet50, MobileNetV2 and YOLOv11) should not be treated as firmly established. Reporting mean and standard deviation across multiple seeds would place the comparison on a firmer footing and is the appropriate next step.

Future work should include:

- Re-evaluating LungCNET on a dataset that preserves patient identifiers, allowing a patient-level split in which no patient contributes images to more than one partition;
- Testing on a larger, more demographically diverse dataset, and on scans acquired with different equipment and protocols;
- Evaluating on a larger benign sample, sufficient to distinguish model performance on the minority class with reasonable confidence;
- Prospective clinical validation with radiologists in the loop.

These results position LungCNET as a candidate tool for further development in lung cancer diagnosis, contingent on the validation steps above. Its consistency across diagnostic categories, and in particular its performance on the benign class, is the property most worth testing on independent data.

## Figures and Tables

**Table 1:** Comparative analysis of deep learning architectures for lung cancer detection across diverse datasets from recent studies.

Ref.	Architecture	Dataset	Performance	Key Findings
[24]	GoogLeNet	IQ-OTH/NCCD	94.38% accuracy	Pre-trained network with lung region extraction preprocessing, yielding enhanced accuracy.
[25]	VGG-16	IQ-OTH/NCCD	98% accuracy	Comparative evaluation of GoogLeNet and VGG16, showing robust cancer detection.
[26]	DCNN	BioMed Research International	71% accuracy	Custom DCNN achieving pathologist-comparable results.
[27]	LCP-CNN	NLST, LUCINDA	94.5% AUC, 99% sensitivity	Specialised architecture with high-performance nodule classification.
[28]	Ensemble (VGG-16, ResNet50, InceptionV3, EfficientNetB7)	IQ-OTH/NCCD + carcinoma dataset	92.8% accuracy	Multi-model ensemble approach for enhanced detection.

**Table 2:** Validation metrics at the early-stopping checkpoint, recorded during the initial training runs under the earlier 80/20 train/validation configuration. Shown to describe convergence behaviour only; all performance results are reported on the held-out test partition in Table 3, Table 4, Table 5 and Table 6.

Model	Validation Accuracy (%)	Best Validation Loss
LungCNET	99.09	0.0268
VGG16	91.36	0.2198
ResNet50	50.91	0.9138
InceptionV3	86.36	0.4738
MobileNetV2	76.36	0.6221
YOLOv11	91.4	0.5784

**Table 7:** Total and trainable parameter comparison. Total parameters reflect each model’s architecture as used in this study (pre-trained backbone, original classification head removed); trainable parameters reflect this study’s partial-unfreezing fine-tuning setup.

Model	Total Parameters (M)	Trainable Parameters (M)
LungCNET	59.21	59.21
ResNet50	23.59	4.47
InceptionV3	21.81	1.94
VGG16	14.72	7.08
MobileNetV2	2.26	0.74
YOLOv11	1.53	1.53

**Table 8:** Inference time (latency) comparison across models.

Model	Single Image (ms)	Batch Processing (ms)
LungCNET	55.76	95.41
VGG16	76.21	242.24
ResNet50	70.83	203.64
InceptionV3	110.85	172.14
MobileNetV2	71.24	109.75
YOLOv11	1.65	0.692

## Data Availability

The dataset supporting this study is publicly available: the IQ-OTH/NCCD Lung Cancer Dataset (Version 2) [33], DOI: https://doi.org/10.17632/bhmdr45bh2.2 (accessed on 11 Auguest 2026). The LungCNET model architecture, training code, and data augmentation pipeline used to produce the results reported in this study are publicly available at: https://github.com/elhamum/LungCNet-revision (accessed on 11 Auguest 2026).
